# Regulation of Tumor Vascular Microenvironment by Nestin and Fms-related Tyrosine Kinase 1 (FLT1) and Their Prognostic Significance in Renal Cell Carcinoma

**DOI:** 10.30699/ijp.2024.2024190.3266

**Published:** 2024-03-03

**Authors:** Noha Elkady, Reham Ahmed Abdelaziz, Rasha Adel Abdelmoneum, Ahmed S Ghonaimy, Dina Mohamed Allam

**Affiliations:** 1 Department of Pathology, Faculty of Medicine, Menoufia University, Shibin Elkom, Egypt; 2 Department of Clinical Oncology and Nuclear Medicine**, **Faculty of Medicine, Menoufia University, Egypt; 3 Department of Urology, Faculty of Medicine, Menoufia University, Shibin Elkom, Egypt

**Keywords:** FLT1, MVD, Nestin, Prognosis, RCC

## Abstract

**Background & Objective:**

Hypervascularity is a characteristic feature of renal cell carcinoma (RCC) and microvessel density (MVD) predicts tumor metastasis. Nestin is a stem cell marker that is expressed in proliferating endothelial cells and newly formed vessels and Fms-related tyrosine kinase 1 (FLT1) is a proangiogenic factor. This study aimed to evaluate the expression of Nestin and FLT1 in RCC and their prognostic impact.

**Methods:**

This retrospective study included sixty cases of RCC after obtaining ethical approval. Sections were immunohistochemically stained by Nestin and FLT1 then their expressions were compared to different clinicopathological parameters. MVD was evaluated using Nestin and CD34 and compared to the different parameters.

**Results:**

Nestin was expressed mainly in endothelial cells of small vessels in 65% of cases while FLT1 was expressed in tumor and endothelial cells in 73.3% of cases. Their expressions were significantly associated with aggressive tumor parameters including larger tumors, high-grade tumors, wider tumor extension, and advanced stage. Moreover, Nestin expression was significantly associated with metastasis. MVD evaluated by Nestin showed more associations with larger tumors, high-grade tumors, wider tumor extension, advanced stage, and metastasis than MVD measured by CD34. Nestin and FLT1 positivity and high MVD measured by Nestin were significantly associated with short overall survival.

**Conclusion:**

Nestin and FLT1 expressions in RCC may be associated with aggressive tumor features and short patients’ overall survival. MVD evaluated by Nestin may be correlated with tumor progression and metastasis. Nestin and FLT1 may be used as prognostic biomarkers in RCC.

## Introduction

Angiogenesis is an integral feature in renal cell carcinoma (RCC) and is supposed to be associated with tumor metastasis. Aberrant angiogenesis in RCC makes the tumor more aggressive and resistant to therapy which makes it a potential target for therapy (1, 2). Previous studies investigated the role of von Hippel–Lindau mutation, hypoxia-inducing factor (HIF), and vascular endothelial growth factor (VEGF) (3) however, limited ones investigated the role of other proangiogenic molecules such as Nestin and FLT1 in RCC.

In addition, previous studies evaluated different methods to assess the degree of angiogenesis, including microvascular density (MVD); however, there is controversy about the best method for assessing MVD. The degree of tumor angiogenesis based on MVD has been proven as a useful predictor of survival in several cancers and is usually associated with a poor prognosis. (4) However, MVD is not an accurate predictor in RCC, while the presence of a higher proportion of immature microvessels was found to be correlated with aggressive cancers and worse prognosis. This arouses the need for markers that identify newly formed immature vessels that may be correlated with RCC prognosis. (5)

Previous literature has investigated numerous therapeutic and prognostic biomarkers that are involved in the molecular mechanism of urinary tract tumors like VEGF, CXCR3 in renal cell carcinoma, PSA in prostate cancer, and other tumors such as IL-17 in CLL (6-9).

Nestin and Fms-related tyrosine kinase 1 (FLT1) are among the suggested prognostic markers in RCC that have not been extensively studied. Nestin is an intermediate filament and a marker for cell stemness. It is only expressed in proliferating vascular endothelial (VE) cells indicating neovascularization and associated with poor prognosis of some cancers. (4, 10, 11) FLT1 regulates endothelial cell proliferation, migration, tumor cell proliferation, and metastasis (12, 13, 14).

This encouraged us to investigate Nestin expression; as being a marker of immature vessels; and a proangiogenic factor (FLT1) in RCC, and to evaluate Nestin^+^ MVD and their impact on RCC progression and patients’ survival.

## Material and Methods

After obtaining the ethical approval, all cases diagnosed as renal cell carcinoma (from Jan 2017 to Jun 2022) were retrieved from the pathology archive of the faculty of Medicine Menoufia University, Menoufia. The cases fulfilling the inclusion criteria were included in the study. The Inclusion criteria included: all cases with available clinical data and available paraffin tissue blocks for re-cut. Exclusion criteria include patients with double primaries, unavailable clinical data, or tissue blocks and blocks not suitable for immunostaining. Sixty cases were included in this retrospective study. The paraffin blocks were collected; then hematoxylin and eosin (H&E) stained slides were examined for evaluation of some histopathological features such as histopathological type, tumor grade using a 2-tiered Fuhrman grading system (15), and tumor extent. Lymph node involvement and pathological stage according to TNM staging (for statistical purposes, stages I and II were considered as early stage while stages III and IV were considered as late stage) were also evaluated. Patients’ sex, age, tumor size, metastasis, follow-up, and overall survival data were collected from patients’ records. 

**Tissue Microarrays**

The examined H&E-stained slides were marked at different areas representing the tumor then the corresponding sites on the blocks were also marked then 3 cores (2mm) were taken from each block using a manual tissue array needle (Beecher Instruments, Silver Spring, USA) and were arranged in the recipient block. A map was drawn to identify the origin of each tissue core. 

**Immunohistochemical Staining**

Sections were cut from each TMA block and were immunohistochemically stained by Nestin, FLT1, and CD34 antibodies using a streptavidin-biotin amplified system, detection kit (Envision, FLEX, code 8002, Dako), and diaminobenzidine (DAB) as a chromogen. Nestin antibody (mouse monoclonal, cat# 3888M18, 7 ml, ready to use, Cell Marque, California, USA) and FLT1 antibody (rabbit polyclonal, cat# RP077-05, 0.5 ml concentrated, Diagnostic BioSystems, Pleasanton, CA) and CD34 antibody (mouse monoclonal, IHC034-100, 0.1 mL concentrated, GenomeMe, CA) were used. Positive and negative control slides were included in each run.

**Immunohistochemical Staining Assessment**

The expression of Nestin and FLT1 was evaluated by two expert pathologists. Expression was assessed in both endothelial and the tumor cells using a two-point scale of the reaction intensity, depending on the resulting color reaction (i.e., 0 for no reaction, indicating negative expression, and 1 for a brown staining reaction, indicating positive expression) considering the subcellular localization of the brown staining. Semi-quantitative scoring of both markers using H-score was also done. The intensity of staining was scored by “H-score” as follows: 0 (no staining); 1 (faint light brown); 2 (pale brown), and 3 (dark brown). The percentage of positively stained cells was also evaluated. The H-score was determined by multiplying the above two scores. Then the expression was divided into two categories: low and high relative to the mean value of the H score**. **(16)

**Assessment of Microvessel Density (MVD)**

MVD was manually assessed in Nestin-stained and CD34-stained slides, where a single blood vessel appeared as a brown-stained endothelial cell cluster. First, the areas of high vascularity (‘hot spots’) were determined by screening the slide at x10 magnification then the MVD was counted in the highest five vascularized fields at x20 magnification then the mean value was calculated. The MVD was divided into low and high using the mean value as a cutoff point (17). 

**Statistical Analysis**

The collected and generated data were tabulated followed by statistical analysis using SPSS version 22 (SPSS Inc., Chicago, Il., USA). Fisher’s exact test (FE), Chi-square test (χ^2^), Mann-Whitney test (U), Kruskal-Wallis (K), and Spearman correlation (r) tests were used. Statistical analysis of the overall survival using a Log-rank test was also done. A P-value ≤ 0.05 was considered statistically significant.

## Results

The mean age of the included 60 cases was 57.4. Males represented 63.3% of the cases. Clear cell type represented 60% of the cases, 13.3% were of papillary type and other types (chromophobe, RCC NOS) represented the remaining 26.7%. Majority of the cases were low grade, limited to the kidney, and presented at an early stage while only two cases were associated with lymph node metastasis and 18.4% of the cases were correlated with metastasis (Table 1).

**Table 1:** Clinicopathological data of the included cases (n= 60)

Clinicopathological parameters	Number (%)
Gender	
Male	38 (63.3%)
Female	22 (36.7%)
Tumor size	
<7	30(50%)
>7	30(50%)
Histopathological types	
Clear	36(60%)
Papillary	8(13.3%)
Others	16(26.7%)
Grade	
Low	38(63.3%)
High	22(36.7%)
Tumor Extent	
T1	22(36.7%)
T2	21(35%)
T3	15(25%)
T4	2(3.3%)
Lymph node	
Negative	58(96.7%)
Positive	2(3.3%)
Pathological Stage	
Early	42(70%)
Advanced	18(30%)
Metastasis	
Negative	40(81.6%)
Positive	9(18.4%)

**Immunohistochemical Results**

**Fig. 1:**
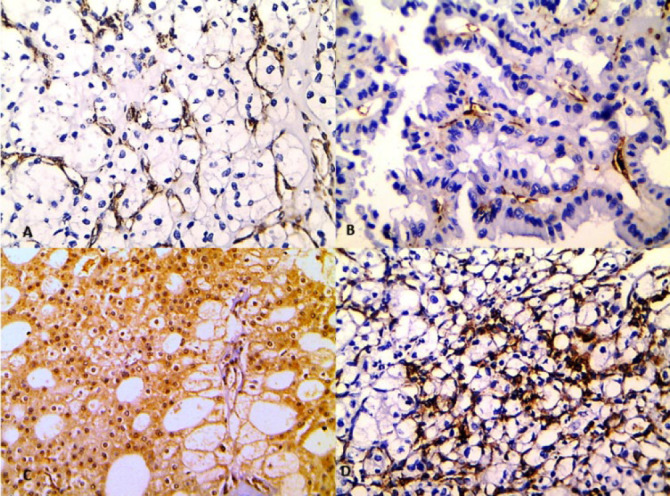
Immunohistochemical (IHC) expression in the renal cell carcinoma. (A) Positive expression of Nestin in the endothelial cells of small vessels in clear RCC. (B) Positive expression of Nestin in the endothelial cells of small vessels in papillary RCC. (C) FLT1 positive expression in the tumor cells and endothelial cells. (D) CD34 positivity in vascular endothelial cells. (IHC X 200).

Nestin positivity was detected in 65% of the RCC cases where it appeared as brown cytoplasmic staining in the endothelial cells of small newly formed capillaries and few scattered tumor cells. FLT1 positivity was observed in 73.3% of RCC cases, it appeared as cytoplasmic with occasional nuclear brown staining in endothelial and the tumor cells. CD34 expression was seen as brown cytoplasmic staining in the endothelial cells lining small and large blood vessels (Figure 1). 

The H score of Nestin expression ranged between 0 and 180 with a mean value of 80.1. Nestin expression was divided into low and high using the mean as a cutoff value. H score of FLT1 expression ranged between 0 and 210 with a mean value of 117.8 which was used as a cutoff point for dividing FLT1 expression into low and high.

**Relationship Between** **Nestin and FLT1** **Expressions and the Clinicopathological Data**

Nestin positivity and high expression level showed significant associations with aggressive tumor parameters such as high grade (*P*=0.011 and 0.008), wider tumor extension (*P*=0.001 and 0.011), advanced stage (*P*=0.017 and 0.001), higher MVD (*P*=0.05 and 0.026) and metastasis (*P*=0.045 and 0.013). In addition, a significant association was observed between Nestin positivity and large tumor size (*P*=0.001) (Table 2).

Moreover, statistically significant associations were found between both positive FLT1 expression and high expression level and high tumor grade (*P*=0.032 and 0.002), wide tumor extent (*P*=0.003 and 0.002), advanced tumor stage (*P*=0.032 and 0.001) and high MVD (*P*=0.01 and 0.007). FLT1 positivity was also associated with larger tumor size (*P*=0.007). There was an association between FLT1 expression and metastasis, but it did not reach a significant level (Table 3).

The Spearman test revealed a significant positive correlation between the H scores of Nestin and that of FLT1 expression (r=0.8 and *P*=0.001).

**Table 2:** Relationship between Nestin expression and the clinicopathological factors of the studied cases

Objectives	Nestin expression			Nestin expression		
	**Negative** **(21)** **Number (%)**	**Positive** **(39)** **Number (%)**	**Test** **P-value** **Odd ratio** **CI**	**Low** **(15)** **Number (%)**	**High** **(24)** **Number (%)**	**Test** **P-value** **Odd ratio** **CI**
Gender						
Male	15(71.4%)	23(59%)	FE *P*=0.4 1.7 (0.5-5.1)	6(40%)	17(70.8%)	FE *P*=0.09 0.3 (0.07-1.1)
Female	6(28.6%)	16(41%)		9(60%)	7(29.2%)	
Tumor Size						
<7	17(81%)	13(33.3%)	FE *P*=0.001* 8.5 (2.3-30.4)	7(46.7%)	6(25%)	FE *P*=0.18 2.6 (0.6-10.3)
>7	4(19%)	26(66.7%)		8(53.3%)	18(75%)	
Histologic type						
Clear	11(52.4%)	25 (64.1%)	χ^2^ *P*=0.56 NA	7(46.7%)	18(75%)	χ^2^ *P*=0.14 NA
Papillary	4(19%)	4 (10.3%)		3(20%)	1(4.2%)	
Others	6(28.6%)	10 (25.6%)		5(33.3%)	5(20.8%)	
Grade						
Low	18(85.7%)	20(51.3%)	FE *P*=0.011* 5.7 (1.4-22.5)	12(80%)	8(33.3%)	FE *P*=0.008* 8 (1.7-36.7)
High	3(14.3%)	19 (48.7%)		3(20%)	16(66.7%)	
Tumor extent						
T1	15(71.4%)	7 (17.9%)	χ^2^ *P*=0.001* NA	5(33.3%)	2(8.3%)	χ^2^ *P*=0.011* NA
T2	4(19%)	17 (43.6%)		9(60%)	8(33.3%)	
T3	2(9.5%)	13 (33.3%)		1(6.7%)	12(50%)	
T4	0	2 (5.1%)		0	2(8.3%)	
Lymph Node						
Negative	21(100%)	37 (94.9%)	FE *P*=0.53 0.6 (0.5-0.7)	15(100%)	22(91.7%)	FE *P*=0.51 0.5 (0.5-0.8)
Positive	0	2 (5.1%)		0	2(8.3%)	
Pathological Stage			FE *P*=0.017* 6.6 (1.3-32.4)			
Early	19(90.5%)	23 (59%)		14(93.3%)	9(37.5%)	FE *P*=0.001* 23.3 (2.6-208.6)
Advanced	2(9.5%)	16 (41%)		1(6.7%)	15(62.5%)	
MVD	38.7 ± 8.7	43.8 ± 9.7	U *P*=0.05*	39.5 ± 11.5	46.5 ± 7.4	U *P*=0.026*
Metastasis						
Negative	14 (100%)	26 (74.3%)	FE *P*=0.045* 0.6 (0.5-0.8)	13(100%)	13(59.1%)	FE *P*=0.013* 0.5 (0.3-0.7)
Positive	0	9 (25.7%)		0	9(40.9%)	

FE= Fisher’s Exact

χ^2^ = Chi square

U= Mann-Whitney test

MVD microvessel density

CI confidence interval * Significant

**Table 3:** Relationship between FLT1 expression and the clinicopathological factors of the studied cases.

Objectives	FLT1 expression			FLT1 expression		
	**Negative** **(16)** **Number (%)**	**Positive** **(44)** **Number (%)**	**Test** **P-value** **Odd ratio** **CI**	**Low** **(13)** **Number (%)**	**High** **(31)** **Number (%)**	**Test** **P-value** **Odd ratio** **CI**
Gender						
Male	10(62.5%)	28(63.6%)	FE *P*=1.00 0.9 (0.3-3.1)	6(46.2%)	22(71%)	FE *P*=0.17 0.4 (0.09-1.3)
Female	6(37.5%)	16(36.4%)		7(53.8%)	9(29%)	
Tumor Size						
<7	13(81.3%)	17(38.6%)	FE *P*=0.007* 6.8 (1.7- 27.7)	8(61.5%)	9(29%)	FE *P*=0.08 3.9 (1-15.2)
>7	3(18.8%)	27(61.4%)		5(38.5%)	22(71%)	
Histologic type						
Clear	8(50%)	28(63.6%)	χ^2^ P=0.51 NA	7(53.8%)	21(67.7%)	χ^2^ *P*=0.47 NA
Papillary	2(12.5%)	6(13.6%)		3(23.1%)	3(9.7%)	
Others	6(37.5%)	10(22.7%)		3(23.1%)	7(22.6%)	
Grade						
Low	14(87.5%)	24(54.5%)	FE *P*=0.032* 5.8 (1.1-28.7)	12(22.3%)	12(38.7%)	FE *P*=0.002* 19 (2.1-145.4)
High	2(12.5%)	20(45.5%)		1(7.7%)	19(61.3%)	
Tumor extent						
T1	12(75%)	10(22.7%)	χ^2^ *P*=0.003* NA	7(53.8%)	3(9.7%)	χ^2^ *P*=0.002* NA
T2	3(18.8%)	18(40.9%)		6(46.2%)	12(38.7%)	
T3	1(6.3%)	14(31.8%)		0	14(45.2%)	
T4	0	2(4.5%)		0	2(6.5%)	
Lymph Node						
Negative	16(100%)	42(95.5%)	FE *P*=1.0 0.7 (0.6-0.8)	13(100%)	29(93.5%)	FE *P*=1.0 0.6 (0.5-0.8)
Positive	0	2(4.5%)		0	2(6.5%)	
Pathological Stage			FE *P*=0.032* 9.4 (1.1-78.1)			
Early	15(93.8%)	27(61.4%)		13(100%)	14(45.2%)	FE *P*=0.001* 0.5 (0.3-0.7)
Advanced	1(6.3%)	17(38.6%)		0	17(54.8%)	
MVD	37.4 ± 10.1	44.4 ± 8.8	U *P*=0.01*	39.1 ± 10.1	36.7 ± 7.4	U *P*=0.007*
Metastasis						
Negative	11(100%)	29(76.3%)	FE *P*=0.09 0.7 (o.6-0.9)	10(100%)	19(67.9%)	FE *P*=0.07 0.6 (0.5-0.8)
Positive	0	9(23.7%)		0	9(32.1%)	

FE= Fisher’s Exact

χ^2^ = Chi square

U= Mann-Whitney test

MVD microvessel density

CI= confidence interval

* Significant

**Relationship between MVD and clinico-pathological parameters**

The MVD showed a significant difference when measured by Nestin and CD34. MVD measured by Nestin ranged between 20 and 50 with a mean value of 35.2 while that measured by CD34 ranged between 20 and 60 with a mean value of 42.5. When comparing MVD measured by Nestin, and that measured by CD34, the first one showed more significant associations with larger tumor extent, and advanced tumor stage (*P*=0.001, 0.001 versus 0.006 and 0.009). Moreover, MVD measured by Nestin showed significant associations with larger tumor size, high-grade tumors, and metastasis (*P*= 0.01, 0.001, and 0.001) (Table 4).

**Survival Analysis**

**Fig. 2:**
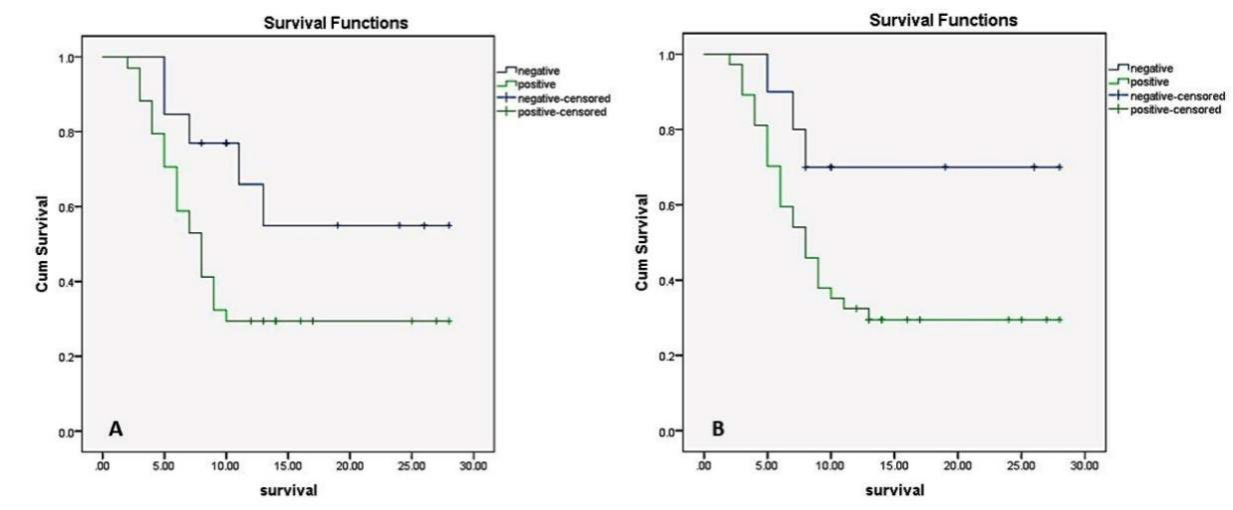
Kaplan Meier analysis of overall survival. Nestin (A) and FLT1 (B) positivity were significantly associated with the shorter patients’ overall survival.

The overall survival of the studied cases was estimated from the date of first diagnosis to the date of last visit or death. It ranged between 2 and 28 months with a mean value of 10.9. Kaplan Meier survival analysis using Log-rank testing revealed that cases with positive Nestin and FLT1 expressions were significantly associated with shorter patients’ overall survival than negative cases (*P*=0.048 and 0.05). (Table 5) (Figure 2). 

High MVD measured by Nestin was significantly associated with the short patients’ overall survival (*P*=0.04) while high MVD measured by CD34 did not affect patients’ survival (*P*=0.6) (Table 5). The Cox proportional hazards regression model revealed that none of the variables was found to be an independent predictor of survival.

**Table 4:** Relationship between MVD measured by both Nestin and CD34 and the clinicopathological factors

Parameters	MVD by Nestin Mean ± SD	Test P value	MVD by CD34 Mean ± SD	Test P-value
Sex		
Male	36.2 ± 7.3	U *P*=0.2	42.4 ± 8.8	U *P*=0.8
Female	33.8 ± 9.1		42.8 ± 11.2	
Size
<7	30.8 ± 6.7	U *P*=0.01*	41.3 ± 9.2	U *P*=0.3
>7	37.5 ± 7.8		43.8 ± 10.1	
Histologic type
Clear	37.5 ± 8.4	K *P*=0.05*	45.4 ± 9.6	K *P*= 0.008*
Papillary	28.6 ± 6.2		37.1 ± 8.4	
Others	32.4 ± 5.4		38.9 ± 8.5	
Grade		
Low	31.8 ± 7.7	U *P*=0.001*	41.7 ± 9.9	U *P*= 0.25
High	38.8 ± 6.9		44.1 ± 9.3	
Tumor extent		
T1	28.2 ± 6.8	K *P*=0.001*	39.7 ± 9.03	K *P*=0.006*
T2	31.7 ± 4.4		41.5 ± 10.8	
T3	41.6 ± 6.3		49.6 ± 4.7	
T4	47.5 ± 3.5		32.5 ±3.5	
Lymph Node		
Negative	34.8 ± 8.1	U *P*=0.2	42.7 ± 9.6	U *P*= 0.5
Positive	42.5 ± 3.5		38.0 ± 11.3	
Pathological Stage		
Early	30.3 ± 5.1	U *P*=0.001*	40.5 ± 9.9	U P=0.009*
Advanced	42.3 ± 6.1		47.5 ± 7.03	
Metastasis				
Negative	32.3 ± 6.5	U *P*=0.001*	41.0 ± 10.1	U P=0.21
Positive	43.3 ± 5.7		45.4 ± 8.7	

SD= standard deviation

U= Mann-Whitney test

K=Kruskal-Wallis test * Significant

**Table 5:** Kaplan Meier's analysis of the overall survival

Parameter	Mean	CI	Test P-value
Nestin expression			
Negative	19.3	13.5-25.1	Log Rank
Positive	12.5	9.1-15.9	0.048
LFT1 expression			
Negative	21.6	15.5-27.6	Log Rank
Positive	12.8	9.5-16.1	0.05
Nestin MVD			
Low	15.6	10.5-20.5	Log Rank
High	6.7	5.1-8.4	0.04
CD34 MVD			
Low	15.1	10.6-19.6	Log Rank
High	13.9	9.6-18.1	0.6

CI confidence interval

## Discussion

RCC is characterized by being a highly vascular tumor where angiogenesis is essentially required for sustained tumor growth and immature newly formed vessels are associated with vascular dissemination. One of the underlying mechanisms of neovascularization in RCC is the stimulation of the proangiogenic factor vascular endothelial growth factor (VEGF) by hypoxia-inducible transcription factor (HIF) (18) however, other mechanisms were not extensively investigated. Our study aimed to investigate the expression of Nestin and FLT1 in RCC and their association with different pathological factors and prognoses.

This study revealed that Nestin and FLT1 expressions in RCC were significantly associated with aggressive tumor parameters. Moreover, Nestin expression was significantly associated with metastasis. It has also been revealed that MVD evaluated by Nestin was associated with RCC progression and metastasis than MVD measured by CD34. Finally, the study has also shown that Nestin and FLT1 positivity and high MVD measured by Nestin were significantly associated with short patients’ overall survival. This has raised attention to the role of Nestin (as a marker of immature vessels) and FLT1 (as a proangiogenic factor) in promoting the growth and progression of RCC. 

The same results were observed in previous studies where Nestin expression was associated with tumor aggressiveness and poor survival rates in glioma and non-small lung cancer (19-21). In addition, Nestin expression was observed to be associated with advanced-stage, metastasis and recurrence in colorectal and liver cancers (22, 23). 

Moreover, these results are in concordance with other studies that described the association between FLT1 expression in colorectal, breast, ovarian, and other tumors and tumor invasion, advanced stage, recurrence, resistance to therapy, and short survival. (13, 24-26) This can be explained by the pro-survival effect of Nestin where it enhances cellular proliferation through regulation of the assembly of microtubules and/or microfilaments, the upregulation of PI3K activity. It also enhances the survival of tumor cells via activation of the Wnt/β-catenin pathway (27).

 It also maintains cellular stemness by induction of CDK5 and Notch1/Hedgehog pathways (28). In addition, Nestin expression promotes tumor invasion via activation of epithelial-mesenchymal transition (EMT) pathways increasing cellular cytoskeleton flexibility (29,30) and induction of integrin-dependent matrix degradation (31).

FLT1 plays a significant role in angiogenesis through binding with high affinity to vascular growth factor A (VEGFA) and placental growth (PlGF). It was found to be overexpressed in endothelial cells of all BV including the proliferating and remodeled vessels. It also regulates vascular sprouting, branching, and assembly of newly formed vessels (32). FLT1 expression was observed in tumor cells of RCC cases. Previous studies have revealed FLT1 expression in tumor cells of breast carcinoma, pancreatic, ovarian, and colorectal carcinoma. FLT1 was detected in tumor cells at the level of mRNA which may suggest that FLT1 is internally expressed in tumor cells. FLT1 works in an autocrine manner while soluble FLT1 can diffuse extracellularly and function in a paracrine fashion (24,25, 33-35). FLT1 expression enhances tumor invasion via activation of placental growth factor (PlGF) and MAPK/metalloproteinase 9 (MMP9), MMP1, and 3 (36, 37), promotion of epithelial-mesenchymal transition (13, 38, 39) and recruitment of metastasis-associated macrophages (40).

This study has also revealed that MVD detected by Nestin could be a better predictor of tumor progression and metastasis than conventional methods. Blood vessels (BV) in RCC are divided into differentiated and undifferentiated ones. Newly formed immature blood vessels are associated with more hematogenous spread because they are leaky and have fragile basement membranes that permit cancer cells to pass into the circulation and spread all over the body, while mature blood vessels have harder impermeable walls that resist cancer cell penetration (41). Nestin is an intermediate filament that maintains stem cell status, and endothelial cell proliferation. Its expression has been detected in proliferating endothelial cells and the origin of these endothelial cells is suggested to be progenitor cells or marrow-derived mesenchymal cells (42,43).

Nestin could be considered as a marker of newly formed vessels whereas CD34 demonstrates all blood vessels including large mature BV. So, the detection of proliferating newly formed BV by Nestin appeared to be a better predictor for tumor metastasis and survival (10).

A positive correlation between Nestin and FLT1 expression was also observed in the studied cases, the same link was recorded in solid tumors where hypoxia-induced factor-1 (HIF-1) expression is elevated in response to the hypoxic environment then HIF-1 induces Nestin and VEGF expression with further activation of FLT1 which enhances tumor angiogenesis.(44,45) Therefore, future investigations should be done to investigate the impact of considering them as biomarkers or the possibility of using them as target therapy in RCC to limit tumor progression and spread and decrease the side effects of anti-VEGF (46, 47).

The absence of funding is the main limitation in this study and the cause for the relatively small sample in addition to the limited availability of research techniques and quality of the equipment which hinder detailed investigation of the role of Nestin and FLT1 in promoting the formation of new immature capillaries and the mechanisms underlying the association between Nestin and FLT1 and RCC progression. Despite that, the results of this study give a preliminary suggestion of the role of Nestin and FLT1 in the formation of new immature capillaries and RCC progression and encourage further wide-scale investigations using more techniques such as in vitro and DNA testing to validate their prognostic roles and investigate the possibility of using them as target therapy instead of conventional antiangiogenic factors.

## Conclusion

Nestin and FLT1 expressions in RCC may be associated with aggressive tumor features and short patients’ overall survival. MVD evaluated by Nestin may be correlated with tumor progression and metastasis. Nestin and FLT1 may be used as prognostic biomarkers in RCC. 
